# Eighteen Months Follow-Up with Patient-Centered Outcomes Assessment of Complete Dentures Manufactured Using a Hybrid Nanocomposite and Additive CAD/CAM Protocol

**DOI:** 10.3390/jcm9020324

**Published:** 2020-01-23

**Authors:** Corina Marilena Cristache, Eugenia Eftimie Totu, Gabriela Iorgulescu, Aida Pantazi, Dorel Dorobantu, Aurelia Cristina Nechifor, Ibrahim Isildak, Mihai Burlibasa, Gheorghe Nechifor, Marius Enachescu

**Affiliations:** 1 Department of Dental Techniques, Faculty of Midwifery and Medical Assisting (FMAM), “Carol Davila” University of Medicine and Pharmacy, 8, Eroilor Sanitari Blvd, 050474 Bucharest, Romania; mburlibasa@gmail.com; 2 Department of Analytical Chemistry, Faculty of Applied Chemistry and Materials Science, University Politehnica of Bucharest, 1-7 Polizu St., sector 1, 011061 Bucharest, Romania; aureliacristinanechifor@gmail.com (A.C.N.); doru.nechifor@yahoo.com (G.N.); 3 Department of Behavioral Science, Faculty of Dental Medicine, “Carol Davila” University of Medicine and Pharmacy, 19 Plevnei Ave., 010221 Bucharest, Romania; gabriela.iorgulescu@umfcd.ro; 4 Center for Surface Science and Nanotechnology (CSSNT), University Politehnica of Bucharest, 313 Splaiul Independentei, District 6, 060032 Bucharest, Romania; aida.pantazi@cssnt-upb.ro (A.P.); dorel.dorobantu@cssnt-upb.ro (D.D.); marius.enachescu@cssnt-upb.ro (M.E.); 5 Department of Bioengineering, Faculty of Chemical and Metallurgical Engineering, Yildiz Technical University, Esenler-Istanbul 34210, Turkey; isildak@yildiz.edu.tr; 6 Academy of Romanian Scientists, Splaiul Independentei 54, 050094 Bucharest, Romania

**Keywords:** PMMA–nanotitania composite, OHIP-EDENT, digital light processing, complete denture, quality of life

## Abstract

The present study aimed to assess the eighteen month follow-up patient-centered outcomes of a simple and predictable protocol for 3D-printed functional complete dentures manufactured using an improved poly(methyl methacrylate) (PMMA)–nanoTiO_2_. A detailed morphological and structural characterization of the PMMA–TiO_2_ nanocomposite, using SEM, EDX, XRD, and AFM, after 3D-printing procedure and post-wearing micro-CT, was also performed. Methods: A total of 35 fully edentulous patients were enrolled in this prospective study. A 0.4% TiO_2_-nanoparticle-reinforced PMMA composite with improved mechanical strength, morphologically and structurally characterized, was used according to an additive computer-aided design and computer-aided manufacturing (CAD/CAM) protocol for complete denture fabrication. The protocol proposed involved a three-step appointment process. Before denture insertion, 1 week, 12 month, and 18-month follow up patients were evaluated via the Visual Analogue Scale (VAS, 0–10) and Oral Health Impact Profile for Edentulous Patients (OHIP-EDENT), with a higher score meaning poor quality of life. Results: A total of 45 complete denture sets were inserted. OHIP-EDENT scored significantly better after 18 months of denture wearing, 20.43 (±4.42) compared to 52.57 (±8.16) before treatment; mean VAS was improved for all parameters assessed. Conclusions: Within the limitations of this study, we can state that the proposed workflow with the improved material used is a viable treatment option for patients diagnosed with complete edentulism.

## 1. Introduction

Digital dentistry has become popular in recent years due to advancements made in the data-acquisition procedures, additive and subtractive manufacturing technologies, and the development of improved materials.

One of the most challenging applications of computer-aided design and computer-aided manufacturing (CAD/CAM) technology is represented by the fabrication of removable complete dentures.

Being the most common and accepted treatment for fully edentulous patients, removable complete dentures manufactured using conventional techniques usually lack fit and resistance due to the resin’s characteristics of polymerization shrinkage, low mechanical resistance, and porosity, among others [1,2]. Several other drawbacks, mostly regarding the skills required from the dental specialist as well as from the dental technician, long chair time, and a great number of patient visits, have led to a new trend towards digital technologies for dentures fabrication [3].

Subtractive manufactured complete dentures, milled out from a pre-polymerized, commercially produced poly(methyl methacrylate) blank, have been claimed to have superior mechanical properties, to exhibit better hygienic properties, and to leach out less residual monomer compared to conventional resin [4], but with increased cost, waste of material (an entire resin blank is used to mill a denture base), and limitations regarding the design of complex geometric shapes. Alternatively, an additive manufacturing process, based on CAD and adding materials directly, layer by layer, is increasingly being used in dentistry. 

Digital light processing (DLP) is used to directly build a structure from 3D CAD data, by exposing photopolymerizable liquid monomer layers to ultraviolet light, and was recently proposed for long-term complete denture manufacturing [5].

To date, in daily practice, the additive manufacturing technique has been solely used for interim complete dentures, due to limited mechanical resistance and stability in time [6], low thermal resistance, reduced antimicrobial action [7], and lack of color stability [8]. 

The extensive use of nanotechnology in dental medicine has opened new options for obtaining materials with improved characteristics [9].

Due to the high demand for rapid, accurate, and resistant material suitable for long-term 3D-printed complete dentures manufacturing, we proposed an improved nanocomposite based on poly(methyl methacrylate) with titanium dioxide nanoparticle inclusions. The newly obtained poly(methyl methacrylate)–TiO_2_ matrix allowed a material with proven antimicrobial action [10], smooth surface aspect [11], and better mechanical and thermal characteristics [12] to be obtained, suitable for use with additive technology [5].

In order to assess the improved hybrid nanocomposite in clinical environment, a protocol for long-term DLP 3D-printed complete denture manufacturing was proposed, and an objective clinical assessment and morphological analysis was performed [13]. In addition to clinical outcomes, it was relevant to evaluate the patients’ subjective perceptions about the treatment using a validated questionnaire. The Oral Health Impact Profile index (OHIP) is a valuable tool for capturing respondents’ perceptions of their oral-health-related quality of life (OHRQoL). The complete OHIP includes 49 questions with seven conceptual domains: functional limitation, pain, psychological discomfort, physical disability, psychological disability, social disability, and handicap, based on the Slade and Spencer model [14]. For edentulous patients, the OHIP-EDENT has been proposed as a shortened, 19 item questionnaire, including all the above-mentioned conceptual domains. OHIP-EDENT is currently considered a valid clinical tool to evaluate fully edentulous patients’ perceptions before and after treatment [15]. 

Despite the high number of studies on digital complete dentures, no published clinical trials to date have reported on patient-centered outcomes with before and after objective evaluation of 3D-printed dentures for long-term wear.

Therefore, the present study aimed to assess the eighteen months follow-up patient-centered outcomes of a predictable and straightforward protocol for 3D-printed functional complete dentures manufactured using an improved poly(methyl methacrylate)–nanoTiO_2_. A detailed morphological and structural characterization of the poly(methyl methacrylate)–TiO_2_ nanocomposite after 3D-printing procedure both before denture delivery and after eighteen months of continuous wear was also performed.

## 2. Experimental Section

### 2.1. Structural Characterization of the Hybrid Nanocomposite Obtained Using Additive Manufacturing–DLP Technology

#### 2.1.1. Materials

The necessary reagents for nanocomposite material preparation were: the base material, commercially available E-Dent 100 (EnvisionTec GmbH, Gladbeck, Germany); a solution containing poly(methylmethacrylate) (PMMA) mixed with polyethylmethacrylate (PEMA) and a radicalic reaction promoter, benzoyl peroxide (BPO), and other additives, named PMMA for simplicity’s sake; titania nanoparticles (TiO_2_) of the anatase variety; benzoyl peroxide; methacrylic acid; and isopropyl alcohol acquired from Sigma Aldrich (Merck, Darmstadt, Germany).

The methacrylic acid was used for nanotitania functionalization prior to its introduction into the PMMA matrix [5]. Thus, it was possible to secure a homogeneous polymeric matrix without nanofiller agglomeration. For the 3D-printing nanocomposite, 0.4% functionalized titania nanoparticles were added to the PMMA solution [12]. 

#### 2.1.2. Methods and Equipment

The polymeric mixtures were obtained with the use of an ultrasonic bath (Elmasonic S10 H, Elma Schmidbauer GmbH, Singen, Germany). The components were weighed on a KERN ALT 220-4NM (Kern & Sohn GmbH, Balingen, Germany) analytical balance.

The obtained nanocomposite material [12] was used for additive complete denture production. Stereolithography–DLP additive manufacturing technology was employed to fabricate the parts in a layer by layer mode, directly from CAD data, using the prepared photoactivated monomer exposed to UV light and polymerized based on the desired final shape. The manufacturing workflow [13] was performed on an EnvisionTEC Perfactory^®^ 3D printer(EnvisionTEC GmbH, Gladbeck, Germany), with layer thickness ranging from 25 µm to 150 µm [16] depending on the settings. The post-processing procedures were as follows: denture soaking in isopropanol for 5 min, drying and removal of printing supports, polishing, final post-curing procedure in a light cure bath (Otoflash G171, EnvisionTEC, Gladbeck, Germany) at 1000 flashes and aesthetic adjustment, as described in detail elsewhere [5,13].

The morphology studies were done through scanning electron microscopy (SEM) analysis using a Hitachi SU 8230 Scanning Electron Microscope (Hitachi High-Technologies Europe GmbH, Mannheim, Germany) equipped with an EDX (energy-dispersive X-ray spectroscopy) Oxford detector-analyzer (Oxford Instruments NanoAnalysis & Asylum Research, Abingdon, UK) for sample composition assessment. Low-angle backscattering and secondary electron images were acquired at different magnifications: 30×, 400×, 1 k×, 10 k×, and 15 k×. 

The morphological and nano-/micro-mechanical properties of the denture teeth sample were studied using a multimodal commercial atomic force microcopy (AFM) system (Solver Next, NT-MDT Co, Moscow, Russia) which was equipped with a nanosclerometric module. Cone-shaped tips of monocrystalline silicon (tip radius ~10 nm) on cantilevers were used to perform the topography measurements, which were successfully carried out at two different scan sizes (10 × 10 µm and 5 × 5 µm) in semi-contact mode.

A sample of 3D-printed denture exposed in a clinical environment for 18 months was studied with the micro-computed tomography technique using high-resolution micro-CT (SkyScan 1272, Brucker, Belgium) equipment. The scanning procedure comprised a 380° rotation, with rotation step set to 0.25° and an average of four frames per slice. A filter of 0.25 mm was applied for scanning. A 70 kV potential and 130 µA intensity was used. The images were acquired at an exposure time per frame of 850 ms, while the image pixel size was set at 7.5 µm. Brucker NRecon software was applied for micro-CT dataset reconstruction.

#### 2.1.3. AFM Investigations–Nanoindentation Studies

The surface roughness, studied using the AFM technique, represents the deviation of an actual sample’s surface topography from an ideal atomically smooth surface [17]. The surface roughness is significant when it is considered as a critical characteristic for material performance, due to the direct influence on the pore size distribution and mechanical resistance for the 3D-printed dentures. 

From the AFM experimental data, namely the acquired topographic images, the root mean square roughness (RMS) and the average roughness (*R_a_*) parameters were calculated using image-processing software [18]. The R_a_ parameter represents the mean value of the surface height relative to the center plane, and it depends on the volumes enclosed by the image of the surface below and above the plane surface area. For its evaluation, the following equation applies:

(1) $$R_{a}=\frac{1}{N}\;\sum_{i=1}^{N}\left|h_{i}-\bar{h}\right|$$

where $\bar{h}$ stands for the vertical height of the surface, h_i_ indicates the mean value of the surface height, and N represents the number of points in the sample area.

The R_a_ parameter provides only the mean absolute profile, without any discrimination between peaks and valleys. Therefore, in the present analysis, when the average surface roughness was relevant, it was recommended to assess other more specific parameters.

Thus, the other parameter, RMS, a statistical measure that represents the standard deviation of h_i_ for the sample area, was considered. Its mathematical expression is given by

(2) RMS= (1N ∑i=1N(hi−h¯)2)12

where $\bar{h}$ indicates the vertical height of the surface, h_i_ is the mean value of the surface height, and N represents the number of points in the sample area.

As a consequence of the RMS roughness’ dependence on square terms, some large deviations from the mean height have a greater influence than the simple difference for the mean roughness. This parameter is much more sensitive to the presence of peaks and valleys than R_a_ due to the squaring of the height of the surface [19].

The determined parameters are very useful when focusing on finding a neat, sharp peak, a possible crack or a scratch that might affect the nanocomposite usage in the oral environment.

For the nanoindentation studies, the equipment was calibrated according to its specificity [20]. All experiments were conducted at room temperature. For each determination (load), the indentations were repeated 10 times, using different areas on the 3D-printed denture surface. The applied loading/unloading rate was 0.1 mN/s. AFM investigation was performed on E-Dent 100 and also on the newly obtained titania nanocomposite material.

### 2.2. Clinical Study Protocol

Thirty-five fully edentulous patients were enrolled in this prospective study, which was performed following the principles and the guidelines of the Helsinki Declaration, revised in 2013 [21]. Bioethical Committee approval from Carol Davila University of Medicine and Pharmacy, Bucharest, Romania, was obtained (no. 98/2016), and the clinical protocol was registered with www.clinicaltrials.gov (ClinicalTrials.govIdentifier: NCT02911038) [13]. 

Inclusion criteria consisted of patients with good local and systemic health; requiring replacement of their maxillary and/or mandibular removable dentures due to tooth wear, denture stains, usage, compromised aesthetics, and function; Class I to III according to American College of Prosthodontists Classification of Complete Edentulism [22]; able to attend all the follow-up sessions and to understand and respond to self-reporting measurement scales and questionnaires; and willing to participate, for at least 18 months, in a follow-up protocol.

Non-compliant patients, patients with severe ridge atrophy, hypertrophic tissues, or oral defects, or patients who disagreed with the CAD/CAM technique for denture manufacturing were excluded. 

Informed consent was obtained from all participants. A 0.4% TiO_2_-nanoparticle-reinforced PMMA composite with low adherence to microbial factors [5], as described in Section 2.1, was used, according to an additive CAD/CAM protocol, for complete denture fabrication. The protocol proposed involved a three-step clinical appointment process as follows. 

The first appointment consisted of data collection and preliminary impressions with an irreversible hydrocolloid (Tropicalgin, Zermack, Badia Polesine, Italy) in stock trays, used to pour a preliminary stone cast for the fabrication of custom impression trays with occlusal rims. 

The second clinical appointment was for functional impressions with Ex-3-N Gold thermoplastic wax (Johannes Meist Dentalfabrikation, Feuchtwangen, Germany), occlusal plane orientation using an occlusal plane indicator, jaw relation records with a gothic arch tracing, using Candulor instrument set for edentulous patients (Candulor AG, Opfikon, Switzerland), tooth mold, shade selection, and anterior tooth positioning guidance. A complete set of intraoral and extraoral photographs were taken at this time, with and without the old dentures and with the adjusted occlusal rims. An Artex face bow (Amann Girrbach AG, Koblach, Austria) was used to transfer the horizontal relationship of the maxillary arch to the cranial base, and data were employed to mount maxillary and mandibular cast in an Artex^®^CR non-Arcon articulator, following the manufacturer’s instructions.

All conventionally obtained data (functional impressions, jaw records, face bow registration) were sent to the laboratory, digitalized using Medit T500 digital scanner (Medit Corp., Seoul, Korea), and imported into EXOCAD^®^ (Exocad GmbH, Darmstadt, Germany) CAD software for complete denture design. The corresponding maxilla–mandibular position and registration were transferred to the Virtual Artex^®^CR in the CAD design software. The anatomical landmarks were identified, and peripheral limits were set on the virtual models. The latter were then aligned according to the clinically captured jaw relation records, and a virtual tooth set-up was performed. For maxillary denture tooth shape and positioning, Planmeca Romexis Smile Design software (Panmeca Oy, Helsinki, Finland) was used. The following average settings, recommended by the manufacturer of the virtual articulator, were considered: sagittal condylar inclination = 30°, Bennett angle = 10°, immediate side shift = 0.5 mm [23,24]. Functionalization of the designed dentures was performed after setting the virtual articulator in occlusal adaptation module and the Cut Intersection tool was utilized to adjust the virtually designed dentures according to the morphology of the antagonists. For bimaxillary edentulous patients, the mandibular denture was functionalized. The CAD program subsequently adapted the tooth morphology dynamically (protrusion, right and left laterotrusion, and retrusion movements), in order to avoid interocclusal interferences.

The virtual set-up (with the digital smile design integrated) was then sent as an electronic preview to the clinician and patient via social media (email, WhatsApp, or Facebook messenger) for approval. A subsequent preview was generated after incorporation of any changes suggested by the clinician. Once the design was approved, the fabrication of the 3D-printed monolithic denture was completed by the laboratory. The detailed workflow used for obtaining the complete dentures via DLP technology and post-processing procedures has been described elsewhere [5]. For denture aesthetic adjustments and customized pink gingiva on the cameo surface, a light-cured Crea.lign veneering system, (Bredent, Senden, Germany) was used [13]. 

At the third patient visit, the dentures were inserted and post-operative instructions provided. Patients were asked to evaluate through Visual Analogue Scale (VAS) the general satisfaction with their dentures. VAS, a psychometric response scale, can be utilized as a useful method to quantify, record, and evaluate qualitative outcomes that are difficult to measure by direct means [17]. The five VAS questions were stated as ‘‘How satisfied are you with your prosthesis considering: aesthetic, speech, masticatory efficiency, hygiene, and comfort?’’ Each question item was measured using a 10 point VAS, with 0 meaning not satisfied at all and 10 meaning completely satisfied. Additionally, self-perception in relation to the impact of oral conditions on physical, psychological, and social wellbeing was evaluated using Oral Health Impact Profile for Edentulous Patients (OHIP-EDENT), validated for the Romanian language (ClinicalTrials.gov Identifier: NCT01392456). Each of the 19 OHIP-EDENT items has a set of possible answers distributed on a Likert scale (4 = always, 3 = frequently, 2 = sometimes, 1 = seldom, and 0 = never), which represents the way that the individual perceives the impact of their oral health. Although the OHIP was originally composed of seven domains (functional limitation, physical pain, psychological discomfort, physical disability, psychological disability, social disability, and handicap), recent studies suggested the concept of four dimensions [25]. Moreover, researchers have considered OHRQoL, summarized by the total score, as meaningful [25,26,27]. In this study, the total score was analyzed, with a value ranging from 0 to 76 and a higher score meaning poorer quality of life.

All participants completed the questionnaires prior to the treatment (baseline, T0), at one week post denture insertion (T1), and at twelve (T12) and eighteen months (T18) follow-up.

Statistical analyses were performed using XLSTAT 2019 (Addinsoft, New York, NY, USA). Changes of parameter values after treatment and over time were investigated using the Mann–Whitney U test. A *p* value < 0.05 was considered significant.

## 3. Results

### 3.1. SEM Investigations

**Figure 1:**
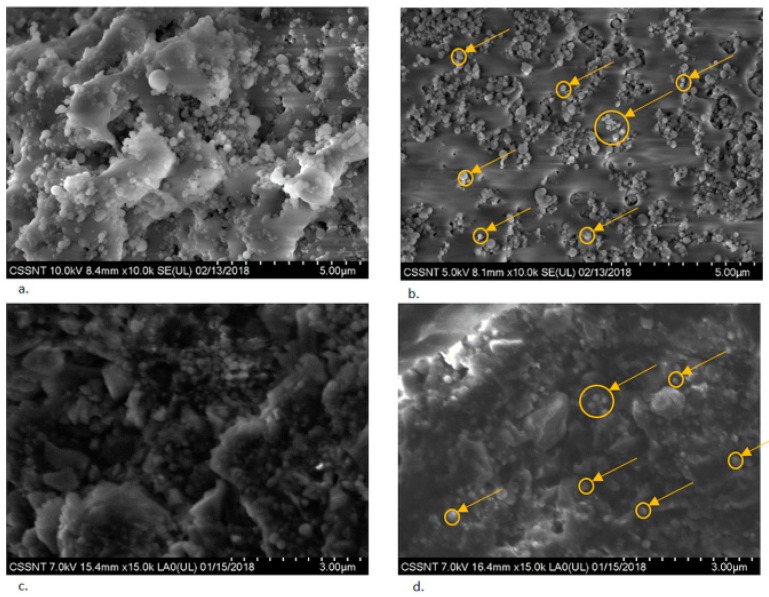
SEM images of (**a**) Dent 100 (not polymerized) and (**b**) newly obtained nanocomposite (not polymerized). Low-angle backscattering electron image of (**c**) 3D-printed E-Dent 100 and (**d**) newly obtained 3D-printed nanocomposite. Titania nanoparticles are marked with yelow circles in subfigures (**b**,**d**).

The images presented in Figure 1 highlight the excellent dispersion of the titania nanoparticles inside the PMMA support. 

**Figure 2:**
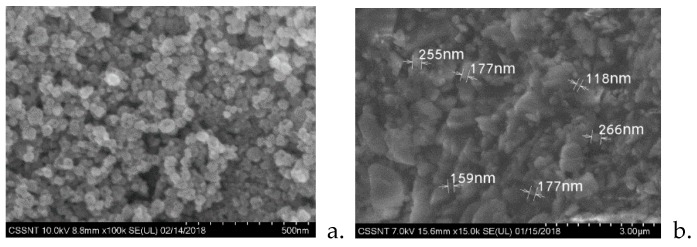
(**a**) SEM images of TiO_2_ powder; (**b**) particle measurements for 3D-printed denture sample.

The nanotitania particles are visualized in Figure 1b,d. Since the nanoparticles were not at all shallow, when a high acceleration voltage was applied, the beam would not penetrate them, and thus the underlying surface could not be reached. Hence, low voltages, 5 kV, or 7 kV were applied to reveal images. The results of the backscattering analysis of the 3D-printed dentures are introduced in Figure 1c,d, while the morphological analysis of the titanium oxide nanoparticles is shown in Figure 2a. 

As shown Figure 1b,d, the improved nanoTiO_2_ composite had a more uniform morphological appearance in its non-polymerized state, as well as in the 3D-printed material, when compared to E-Dent100.

The dimensions of the particles embedded in the 3D-printed polymeric matrix are highlighted in Figure 2b, along with the distribution according to the particle diameters.

**Figure 3:**
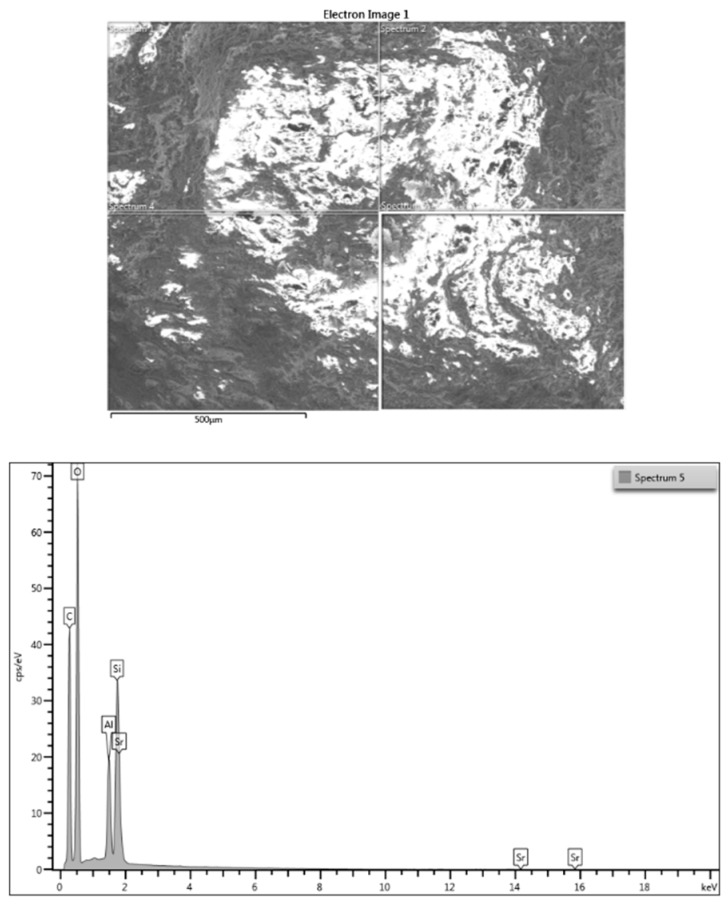
Energy dispersive X-ray (EDX) analysis for 3D-printed E-Dent 100.

**Figure 4:**
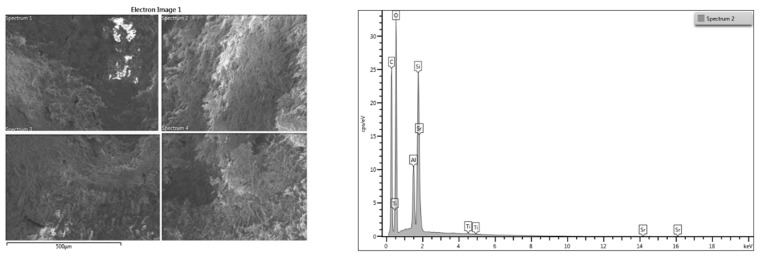
EDX analysis for the newly obtained 3D-printed nanocomposite (denture).

The 3D-printed samples with and without nanotitania were analyzed using energy dispersive X-Ray spectroscopy (EDX) in composition profile mode. The EDX analysis allowed the differences in composition of the samples E-Dent 100 and the newly obtained nanocomposite to be established across different regions of interest, and the elements distribution within the complex matrix to be identified. The EDX results are presented in Figure 3 and Figure 4. 

**Figure 5:**
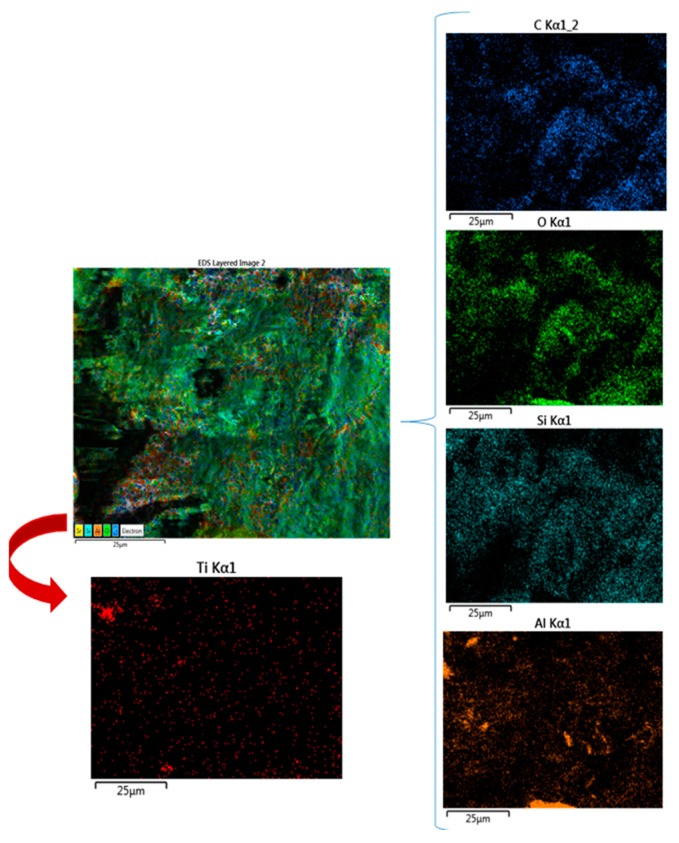
Elemental mapping for the 3D-printed nanocomposite.

By using the capacity of EDX analysis to study a small area, the elemental percentage for the PMMA–nanotitania analyzed sample was determined. The elemental mapping is reproduced in Figure 5.

### 3.2. Nanoindentation–AFM Investigations

**Figure 6:**
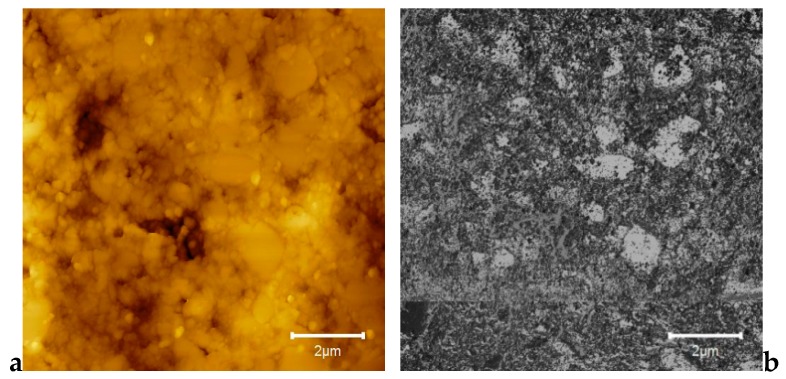
10 × 10 µm 2D atomic force microscopy (AFM) images of the 3D-printed E-Dent 100 sample: (**a**) topography; (**b**) phase contrast.

Some representative AFM images for E-Dent 100, recorded at high magnification of 10 µm × 10 µm, with both topography and phase contrast, are presented in Figure 6a,b. 

**Figure 7:**
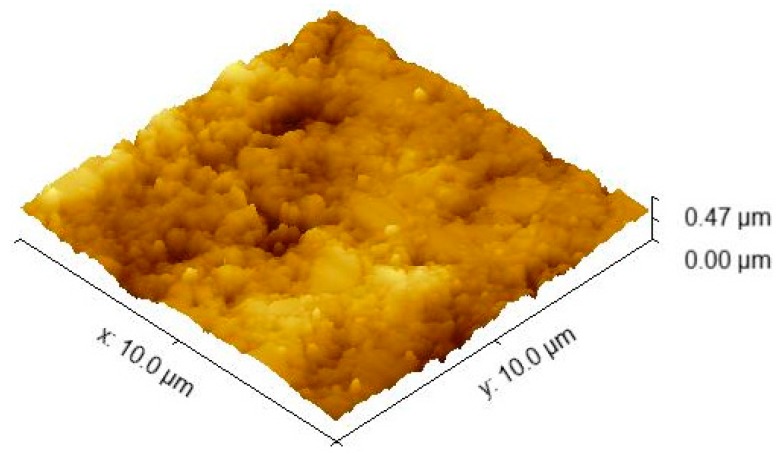
10 × 10 µm 3D AFM image of the 3D-printed E-Dent 100 sample.

In Figure 7, the 3D image captured via the AFM technique is presented for the considered sample size of 10 × 10 µm. 

**Figure 8:**
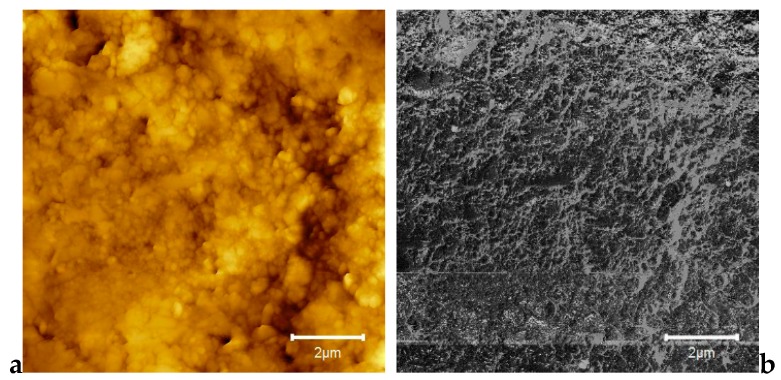
10 × 10 µm 2D AFM images of the 3D-printed nanocomposite with 0.4% nanoTiO_2_: (**a**) topography; (**b**) phase contrast.

**Figure 9:**
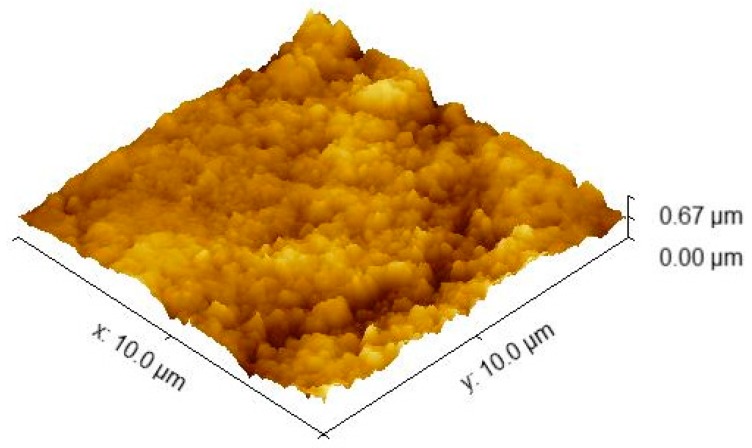
10 × 10 µm 3D AFM image of the 3D-printed newly obtained composite with 0.4% nanoTiO_2._

The AFM images at a high magnification of 10 µm × 10 µm, showing both topography and phase contrast for the 3D-printed composite with 0.4% nanoTiO_2_ are presented in Figure 8a,b. Figure 9 shows the 3D image captured by the AFM technique for the considered sample size of 10 × 10 µm.

Also, a similar 3D topography was taken for a sample size of 5 × 5 µm.

**Figure 10:**
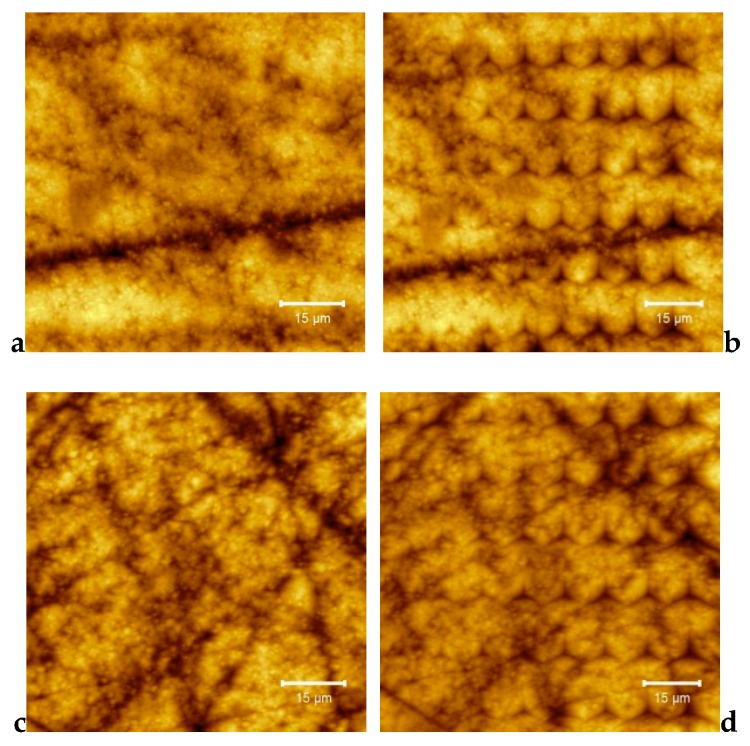
80 × 80 µm and 90 × 90 µm 2D topography images of 3D-printed: (**a**) E-Dent 100 before and (**b**) after performing the indentation curves; (**c**) the newly obtained composite with 0.4% nanoTiO_2_ before and (**d**) after performing the indentation curves.

The hardness, contact stiffness, elastic modulus, or penetration depth are important parameters derived from the nanoindentation studies. The recorded data are based on ten indentations performed on the considered sample (E-Dent 100 and the nanocomposite with 0.4% nanoTiO_2_) are comparatively presented in Figure 10a–d. 

The contact stiffness against penetration depth allowed the stiffness to be calculated from the unloading curve slope for both considered samples (E-Dent 100 and the newly obtained composite with 0.4% nanoTiO_2_). 

**Figure 11:**
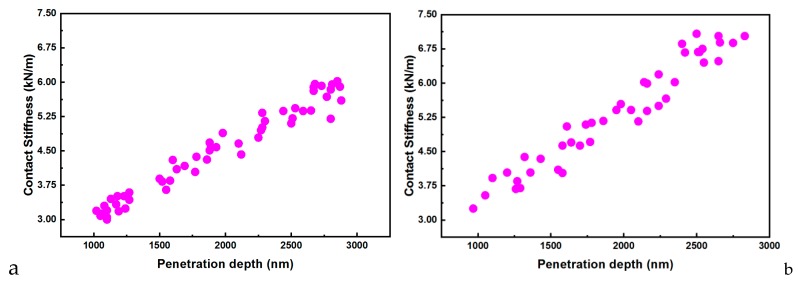
Contact stiffness versus penetration depth for (**a**) E-Dent 100 and (**b**) the newly obtained composite with 0.4% nanoTiO_2._

**Figure 12:**
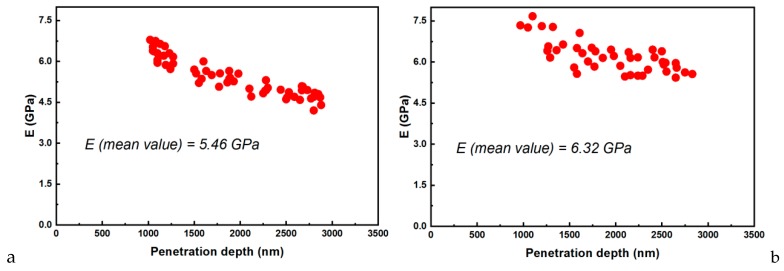
Elastic modulus versus penetration depth for (**a**) E-Dent 100 and (**b**) the newly obtained composite with 0.4% nanoTiO_2_

Regarding the material’s behavior during loading, it was assumed that the deformation was both elastic and plastic. In contrast, for unloading, it was assumed that only elastic deformations were recovered. This elastic behavior for the unloading curve helped with the analysis and permitted the determination of the mechanical characteristics of hardness (H) and elasticity modulus (E) [28]. Thus, E could be determined if the stiffness (S) was found using the slope of the unloading curve (Figure 11). Based on experimental data, the elastic modulus was calculated [29], and its variations for both samples are presented in Figure 12.

**Figure 13:**
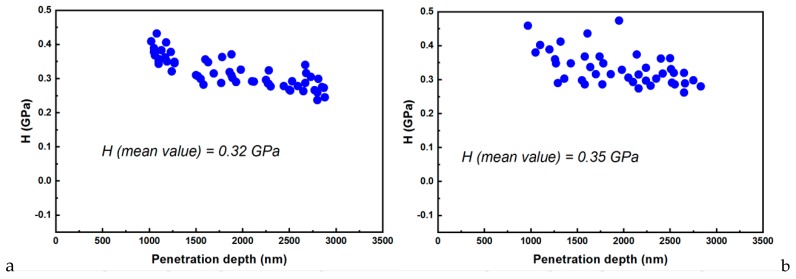
Hardness versus penetration depth for (**a**) E-Dent 100 and (**b**) the newly obtained composite with 0.4% nanoTiO_2._

The hardness was determined as the ratio between the area of contact at peak load and the maximum load [30,31]. The nanoindentation measurements resulted in the hardness values represented in Figure 13.

The elastic modulus (Figure 12) was determined using the experimental data from the loading/unloading curves. This parameter can describe a material’s stiffness, being an essential factor regarding restorative reliability. 

The AFM images presenting the top surface roughness allowed the values of RMA and R_a_ parameters to be extracted. Thus, for the 10 × 10 µm sample, the statistical quantities calculated for the 2D topography image for both samples are presented in Table 1.

When acquiring the data from a sample with dimensions 5 × 5 µm, the RMS and R_a_ values decreased, as expected. The statistical quantities calculated for the 2D topography image (5 × 5 µm) are shown in Table 2. 

Using the recorded data from the nanoindentation measurements (Figure 10), the R_a_ and RMS were calculated, and they are presented in Table 3. 

### 3.3. Clinical Study Results

Of the total 35 patients enrolled in this study, aged 48 to 81 years old, mean 64.26 (±8.27), 23 were females and 12 males. No patients were lost to follow-up at 16 months.

A total of 45 complete edentulous arches (31 maxillary and 14 mandibular) were restored with 3D-printed dentures using DLP manufacturing technology and the improved nanoTiO_2_ polymeric material, according to the protocol described in Section 2.2.

Two dentures were accidentally broken during the first 6 months and reprinted using the stl (stereolithography) files by the dental laboratory, without any other preliminary clinical appointment.

**Figure 14:**
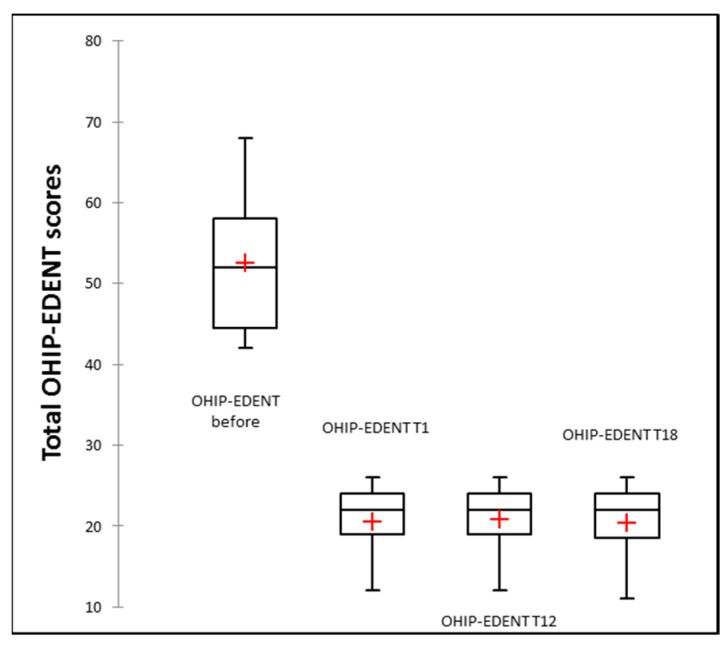
Boxplots for the changes in OHIP-EDENT overall scores.

Mean OHIP-EDENT total scores at baseline (before treatment), at 1 week post denture insertion (T0), and at 12 (T12) and 18 months (T18) are presented in Table 4. Significant reductions in OHIP- EDENT scores for the maxillary, mandible, both restored arches, and for the overall treatment group were registered at the 1 week and 12 and 18 month follow-ups (*p* < 0.05), as shown in Table 4. Additionally, no statistically significant differences were noticed over time (at 18 months as compared to 1 week) with the inserted 3D-printed dentures. The side by side boxplots for the change in OHIP-EDENT overall score are presented in Figure 14.

**Figure 15:**
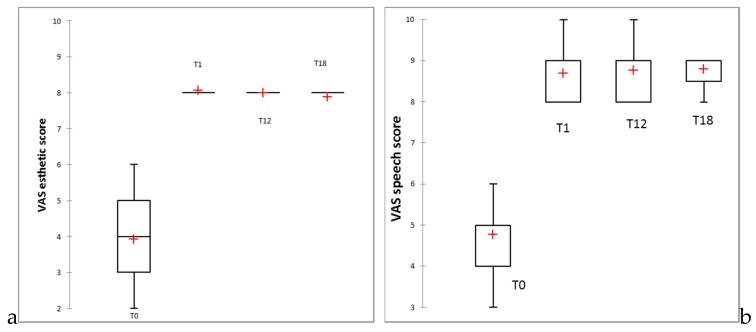

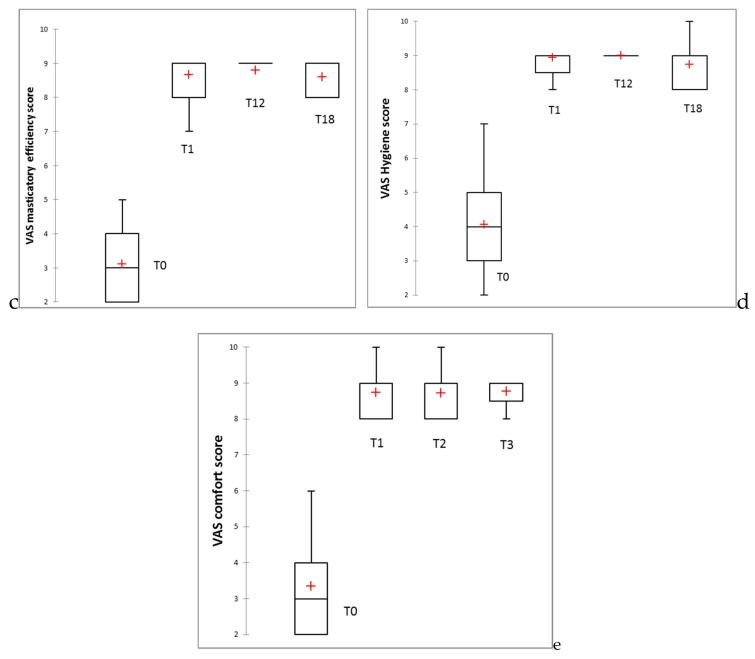
Boxplots for the changes in VAS overall scores: (**a**) aesthetic, (**b**) speech, (**c**) masticatory efficiency, (**d**) hygiene, (**e**) comfort.

The mean values of general satisfaction scores on the evaluated period are provided as Supplementary Material (Supplementary Table S1). Statistical significant improvements in all criteria assessed were recorded. However, the lowest mean value for the 3D-printed dentures was registered at 18 months for aesthetic evaluation (7.86 ± 0.36 for the restored maxilla patients, with 7.89 ± 0.41 for the overall studied group). Despite this, the aesthetic result was evaluated statistically significantly better than before denture insertion (Supplementary Table S1, Supplementary Material) and was not significantly different compared to one week post denture insertion. The side by side boxplots for the changes in general satisfaction scores for aesthetic, speech, masticatory efficiency, hygiene, and comfort are presented in Figure 15a–e.

### 3.4. Micro-CT Post-Wearing Investigations

**Figure 16:**
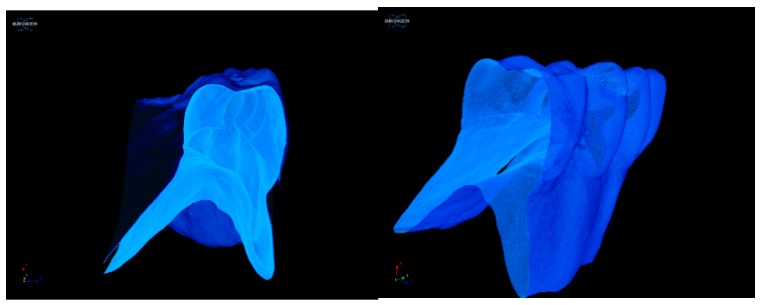
Slice images using micro-CT performed on a sample of the 3D-printed denture.

As the micro-CT technique is a nondestructive analysis, our investigated samples were not modified during the applied procedure. The preservation of the samples was an important aspect, as the samples could be re-tested at a later stage or used for other tests [32,33]. In Figure 16, an image sample of a denture used clinically for 18 months is presented. 

For the 3D-printed denture analysis, a grey-level threshold was applied [34]. Noting that the micro-CT technique through its X-ray absorption is sensitive to material density, we asserted that the area with higher density highlighted in Figure 16 could be assigned to the presence of the added nanotitania in the PMMA matrix. The structure visualized showed a satisfactory uniformity of the nanofiller dispersion inside the polymeric network. Additionally, no defects such as cracks or pores could be identified for the sample of the worn denture (18 months). 

## 4. Discussion

The proposed nanocomposite was obtained using E-Dent 100 as a base material, recommended by the producer to be employed for individual long-term temporary restorations (crowns and bridges with no more than one pontic, in the anterior and posterior region), with DLP equipment. The characteristics of the base material were improved by adding nanotitania, as described in Section 2.1.2. with the aim of alleviating some disadvantages known for PMMA as a denture material [5] and, subsequently, to use it for 3D-printed complete denture manufacturing 

The nanoTiO_2_ hybrid material proved to be adequate for DLP technology, with excellent biocompatibility [10] in the oral environment and with antibacterial properties against *Candida* species [5], the results being presented in previous published papers. 

The present study aimed to perform a detailed morphological and structural characterization of the poly(methyl methacrylate)–TiO_2_ nanocomposite after 3D-printing procedure for complete denture manufacturing, followed by 18 month assessment of patient-centered outcomes.

It is necessary to mention that while other studies have been based on limited structural investigations without correlation with the intrinsic properties of the dental materials for 3D printing, our work provides a framework for combined data analysis, applying various techniques in order to ensure a qualitative estimation of specific properties of the PMMA–nanotitania composite. Our assessment introduced a new perspective for correlating the material’s characteristics with its morphological structure. Furthermore, our study paid attention to specific issues, such as the specific surface area dictated by the nanoparticles embedded in the PMMA matrix.

Although functionalized nanofiller was added to avoid the local agglomerations of the nanoparticles, inhomogeneous area still existed. In this context, concerns were raised regarding the mechanical resistance of the 3D-printed dental devices. Nevertheless, the nanoindentation determination showed that the material presented excellent mechanical strength as compared to the original E-Dent 100.

The comparative elemental analysis (Figure 4) highlighted the presence of Ti in the composition of the 3D-printed material. The presence of the other inorganic additives like Si in both samples was used to improve the mechanical resistance of the material. 

As shown in Figure 5, the elemental maps of the atomic concentrations of Ti revealed that Ti was homogeneously dispersed throughout the PMMA matrix. The general aspect of the images recorded through the AFM technique was almost flat, although some elevated sites occurred due to the titania nanoparticle agglomeration, compared to E-Dent 100 (Figure 6, Figure 7, Figure 8, Figure 9 and Figure 10).

The AFM investigation proved that no phase separation was present, nor were relevant agglomerations of nanoparticles emerging on the surface.

The nanocomposites with higher elastic moduli were better for our intended application, manufacturing 3D-printed complete dentures, as were better able to withstand higher occlusal forces, being able to avoid deformation and fractures of teeth and the denture base. The maximum masticatory force in some people may reach up to 500 to 700 N/cm^2^ [35]. Consequently, the calculated values for E = 6.34 GPa (as compared to 5.46 for E-Dent 100) and for hardness 0.35 GPa (0.32 GPa for E-Dent 100) provided a comfortable mechanical resistance of the 3D-printed nanocomposite to masticatory forces, being suitable for complete denture manufacturing.

A clinical study involving 35 patients was performed for the in vivo assessment of the newly obtained material and its suitability for long-term complete denture fabrication using additive CAD/CAM technology. The retention and stability of complete dentures of the patients, included in this study group, were assessed at 1 week, 6 months, 12 months, and 18 months post denture insertion by two experienced prosthodontists using the modified Kapur index [36,37]. A significant improvement in denture retention and stability was noticed for maxillary and mandibular dentures, and the obtained result was maintained throughout the follow-up period (18 months), the results being presented in a previous published papers [13]. 

The present study evaluated OHIP summary before 3D-printed denture insertion, at 1 week post-insertion and also at 12 and 18 months. Even though there were three types of edentulous patients restored—fully maxillary (21 patients), fully mandibular (4 patients), and both arches (10 patients)—a statistically significant improvement in oral-health-related quality of life (OHRQoL) assessed with OHIP-EDENT was observed, explained by the good retention and stability of the additively manufactured dentures. These results could be thanks to a good denture base adaptation and border seal with minimal distortion during processing with CAD/CAM technology [38]. Moreover, Hyung and co-workers, comparing in vitro the trueness and tissue surface adaptation of mandibular DLP dentures to milled dentures, found similar results, with a slightly better accuracy of milled dentures [39].

Regarding patient satisfaction with the new dentures, a significant improvement in all aspects was noticed, and no essential changes occurred over time. However, the lowest score was found in the appearance satisfaction of the maxillary denture group after 18 months (mean VAS score of 7.80 out of 10; Supplementary Table S1, Supplementary Material). This result could be explained by the color changes of acrylic resins in the oral cavity, mostly due to the slow absorption of water over time, which is a property attributable to the polar nature of the resin molecules [40]. A 1 year comparative assessment of the color changes of the 3D-printed maxillary complete dentures on 10 patients was performed and the color change over a one year period was below the maximum acceptability threshold, in accordance with the patients’ subjective evaluation, who were unable to identify the color change, and three times lower than the original E-Dent 100 [41].

The general improvements in patient satisfaction were in accordance with the study by Katadiyil and co-workers, comparing treatment outcomes from two fabrication techniques (conventional and CAD/CAM) [35]. They reported that patients revealed significantly higher overall patient satisfaction with CAD/CAM complete dentures.

In spite of the fact that conventionally manufactured removable complete dentures are still considered the standard of care for restoring fully edentulous patients [42], and that treatment with complete dentures presents a positive impact on patients’ OHRQoL [43], previous research has shown that these dentures deform during processing, leading to diminished retention, stability, and support, with adverse consequences for the patient’s comfort and increasing clinician chair time because of required adjustments [38,44]. 

Although the presented partially digital workflow proposed for complete denture manufacturing has numerous advantages, including a reduced clinical chair time (three appointments) for denture fabrication and placement, the ability to provide a replacement or a spare prosthesis by using the stored digital data, the possibility of obtaining functionalized dentures by using the virtual articulator in prosthesis design, and reduced costs and loss of material when comparing to CAD/CAM milling dentures, several improvements, mainly regarding the aesthetic appearance, are still needed. 

The proposed protocol for complete denture manufacturing was shown to be feasible, and removable prosthetic devices that met patients’ requirements could be predictably fabricated. No significant changes in patient satisfaction were observed after 18 months of continuous wearing, and no defects such as cracks or pores were identified when denture samples were investigated using a non-destructive micro-CT technique.

The present study had several limitations, such as the lack of a control group with conventionally manufactured dentures and the lack of comparison with CAD/CAM milled dentures. Additionally, material compatibility with conventional relines and the changes in the surface adaptation over time in saliva immersion need to be evaluated.

## 5. Conclusions

Within the limitations of this 18 month study, we drew the following conclusions.

- The proposed workflow with the nanoTiO_2_ composite material used is a viable treatment option for patients diagnosed with complete edentulism.
- The results indicate that the OHRQoL was significantly improved, and patients’ satisfaction scores for aesthetic, speech, masticatory efficiency, hygiene, and comfort were significantly higher upon new denture insertion, with these improvements being maintained throughout the entire evaluation period.
- The PMMA nanocomposite with 0.4% TiO_2_ facilitated the manufacturing of performant 3D-printed complete dentures, which maintained their improved characteristics after permanent usage by patients for 18 months.

## 6. Patents

“Nano-composite material used for making dental prosthesis by stereolithography comprises poly(methyl methacrylate) and titanium dioxide nanoparticles and has uniform morphological structure and antibacterial action without cytotoxicity” Patent pending no.R0132968·AO, Derwent Manual Code(s): A04-F06ES; AOS·C; All- 816; All-C02; Al2-V028; AU-Wl4; 008-A03; 009-AOlA; D09-C 01; P 32·MOB; P32·M.

## Figures and Tables

**Table 1:** Statistical quantities calculated for the 2D topography image (10 × 10 µm).

	E-Dent 100	PMMA with 0.4% nanoTiO_2_
**R_a_**	40.3 nm	59.1 nm
**RMS**	52.4 nm	77.8 nm

R_a,_ average roughness; RMS, root mean square roughness; PMMA, poly(methyl methacrylate).

**Table 2:** Statistical quantities calculated for the 2D topography image (5 × 5 µm).

	E-Dent 100	PMMA with 0.4% nanoTiO_2_
**R_a_**	21.3 nm	25.9 nm
**RMS**	28.5 nm	34.6 nm

R_a,_ average roughness; RMS, root mean square roughness; PMMA, poly(methyl methacrylate).

**Table 3:** Statistical quantities calculated for the 2D topography images (80 × 80 µm and 90 × 90 µm, respectively) recorded before and after performing the indentation curves.

	R_a_ (nm)		RMS (nm)	
	BEFORE	AFTER	BEFORE	AFTER
E-Dent 100	98.7	105.9	125.7	131.7
PMMA with 0.4% nanoTiO_2_	88.3	96.3	107.5	119.7

R_a,_ average roughness; RMS, root mean square roughness; PMMA, poly(methyl methacrylate).

**Table 4:** Mean and standard deviation of total OHIP-EDENT scores post denture insertion (T0) and at 12 (T12) and 18 months (T18). Comparison between baseline and 18 months evaluation, respectively, 1 week follow-up and 18 months.

Patients According to Restored Arches	Mean (Standard Deviation) of Total OHIP-EDENT Scores				*p* (T0 and T18)	*p* (T1 and T18)
	T0	T1	T12	T18		
Maxilla (*n* = 21)	52.62 (8.70)	20.62 (4.51)	20.81 (4.37)	20.33 (4.27)	*p* ≤ 0.00	0.75
Mandible (*n* = 4)	56.50 (6.66)	21.25 (2.99)	21.25 (2.99)	22.25 (3.10)	0.02	0.76
Both arches (*n* = 10)	50.90 (7.67)	20.10 (5.22)	20.40 (4.84)	19.90 (5.32)	*p* ≤ 0.00	1.00
Overall (*n* = 35)	52.57 (8.16)	20.54 (4.48)	20.74 (4.27)	20.43 (4.42)	*p* ≤ 0.00	0.89

Statistical significance: *p* < 0.05.

## Supplementary Materials

The following are available online at https://www.mdpi.com/2077-0383/9/2/324/s1, Table S1. Mean and standard deviation of VAS scores post dentures insertion (T0) and at 12 (T12) and 18 months (T18). Comparison between baseline and 18 months evaluation.
