# Supplementary material for: Eighteen Months Follow-Up with Patient-Centered Outcomes Assessment of Complete Dentures Manufactured Using a Hybrid Nanocomposite and Additive CAD/CAM Protocol
Source: J Clin Med. 2020 Jan 23;9(2):324. doi: 10.3390/jcm9020324 (PMC7073708; doi:10.3390/jcm9020324)
Supplement: Supplementary file 1 [file jcm-09-00324-s001.pdf]

Supplementary Material

Table S1. Mean and standard deviation of VAS scores post dentures insertion (T0) and at 12 (T12) and 18 months (T18). Comparison between baseline and 18 months evaluation.

VAS	Mean (standard deviation) of VAS scores												P (T0 and T18)			
	T0				T1				T18							
	Max (n=21)	Mand (n=4)	Max&Mand	All (n=35)	Max(n=21)	Mand(n=4)	Max&Mand	All (n=35)	Max(n=21)	Mand(n=4)	Max&Mand	All (n=35)	Max(n=21)	Mand(n=4)	Max&Mand	All (n=35)
Esthetic	3.81 (1.08)	4.00 (0.82)	4.10 (1.10)	3.91 (1.04)	8.10 (0.44)	8.25 (0.96)	7.90 (0.57)	8.06 (0.54)	7.86 (0.36)	8.25 (0.96)	7.80 (0.42)	7.89 (0.41)	p ≤ 0.00	0.02	p ≤ 0.00	p ≤ 0.00
Speech	4.90 (0.94)	4.75 (0.96)	4.50 (0.85)	4.77 (0.91)	8.67 (0.66)	8.75 (0.50)	8.70 (0.48)	8.69 (0.58)	8.81 (0.60)	8.75 (0.50)	8.80 (0.42)	8.80 (0.53)	p ≤ 0.00	0.02	p ≤ 0.00	p ≤ 0.00
Masticatory efficiency	3.10 (1.04)	3.00 (0.82)	3.20 (1.14)	3.11 (1.02)	8.71 (0.56)	8.75 (0.50)	8.50 (0.71)	8.66 (0.59)	8.57 (0.51)	8.75 (0.50)	8.60 (0.52)	8.60 (0.50)	p ≤ 0.00	0.02	p ≤ 0.00	p ≤ 0.00
Hygiene	3.57 (1.43)	5.50 (0.58)	4.50 (1.27)	4.06 (1.46)	8.95 (0.74)	9.00 (0.82)	8.90 (0.57)	8.94 (0.64)	8.67 (0.48)	8.75 (0.50)	8.90 (0.57)	8.74 (0.51)	p ≤ 0.00	0.02	p ≤ 0.00	p ≤ 0.00
Comfort	3.57 (1.08)	2.75 (0.96)	3.10 (1.29)	3.34 (1.04)	8.76 (0.54)	9.00 (0.00)	8.60 (0.52)	8.74 (0.51)	8.81 (0.51)	8.75 (0.50)	8.70 (0.48)	8.77 (0.49)	p ≤ 0.00	0.02	p ≤ 0.00	p ≤ 0.00

Statistical significance, $P < 0.05$.
